# Evaluating the impact, implementation experience and political economy of primary care networks in Kenya: protocol for a mixed methods study

**DOI:** 10.1186/s12961-024-01273-w

**Published:** 2025-01-27

**Authors:** Beatrice Amboko, Jacinta Nzinga, Benjamin Tsofa, Peter Mugo, Anita Musiega, Beryl Maritim, Ethan Wong, Caitlin Mazzilli, Wangari Ng’ang’a, Brittany Hagedorn, Gillian Turner, Anne Musuva, Felix Murira, Nirmala Ravishankar, Salim Hussein, Edwine Barasa

**Affiliations:** 1 Health Economics Research Unit, KEMRI-Wellcome Trust Research Programme, Nairobi, Kenya; 2 Liverpool School of Tropical Medicine, Liverpool, United Kingdom; 3 Bill and Melinda Gates Foundation, Seattle, United States of America; 4 ThinkWell, Global, Nairobi, Kenya; 5 Division of Primary Health Care, Ministry of Health, Nairobi, Kenya; 6 Nuffield Department of Medicine, University of Oxford, Oxford, United Kingdom

**Keywords:** Primary care networks, Primary health care, Impact evaluation, Process evaluation, Political economy analysis, Gender analysis, Kenya

## Abstract

**Background:**

Primary care networks (PCNs) are increasingly being adopted in low- and middle-income countries (LMICs) to improve the delivery of primary health care (PHC). Kenya has identified PCNs as a key reform to strengthen PHC delivery and has passed a law to guide its implementation. PCNs were piloted in two counties in Kenya in 2020 and implemented nationally in October 2023. This protocol outlines methods for a study that examines the impact, implementation experience and political economy of the PCN reform in Kenya.

**Methods:**

We will adopt the parallel databases variant of convergent mixed methods study design to concurrently but separately collect quantitative and qualitative data. The two strands will be mixed during data collection to refine questions, with findings triangulated during analysis and interpretation to provide a comprehensive understanding of PCN implementation. The quantitative study will use a controlled before and after study design and collect data using health facility and client exit surveys. The primary outcome measure will be the service delivery readiness of PHC facilities. We will use a random sample of 228 health facilities and 2560 clients in four currently implementing PCNs, four planning to implement and four control counties at baseline and post-implementation. We shall undertake a preliminary cross-sectional analysis of the data at baseline from October to December 2023, followed by a difference-in-difference analysis at the endline from October to December 2024 to compare the outcome differences between the intervention and control counties over a 12-month period. The qualitative study will include a cross-sectional process evaluation and political economy analysis (PEA) using document reviews and approximately 80 in-depth interviews with national and sub-national stakeholders. The process evaluation will assess the emergence of PCN reforms, the implementation experience, the mechanism of impact and how the context affects implementation and outcomes. The PEA will examine the interaction of structural factors, institutions and actors/stakeholders’ interests and power relations in implementing PCNs. We will also examine the gendered effects of the PCNs, including power relations and norms, and their implications on PHC from the supply and demand sides. We shall undertake a thematic analysis of the qualitative data.

**Discussion:**

This evaluation will contribute robust evidence on the impact, implementation experience, political economy and gendered implications of PCNs in a LMIC setting, as well as guide the refining of PCN implementation in Kenya and other LMICs implementing or planning to implement PCNs to enhance their effectiveness.

## Background

Kenya has pledged to achieve universal health coverage (UHC) by 2030 [1]. UHC means everyone can access high-quality health care services without being impoverished [2]. Primary health care (PHC) has been identified as a key foundation for Kenya’s UHC ambitions [1]. PHC is essential care accessible to individuals and communities in acceptable ways, through their full participation, and at an affordable cost to the community and the country [3]. PHC is a whole-of-society and whole-of-government approach to health that combines the following three components: essential public health functions and primary care as the foundation of integrated health services, empowered people and communities, and multisectoral policy and action [4, 5]. The aim of PHC is to ensure an equitable distribution of the highest possible level of health and wellbeing, focusing on the needs of the people early on along the health continuum from the promotion of health and prevention of diseases to treatment, rehabilitation and palliative care, and as close as possible to the people [4]. PHC is an efficient and equitable way to achieve UHC [6].

**Fig. 1:**
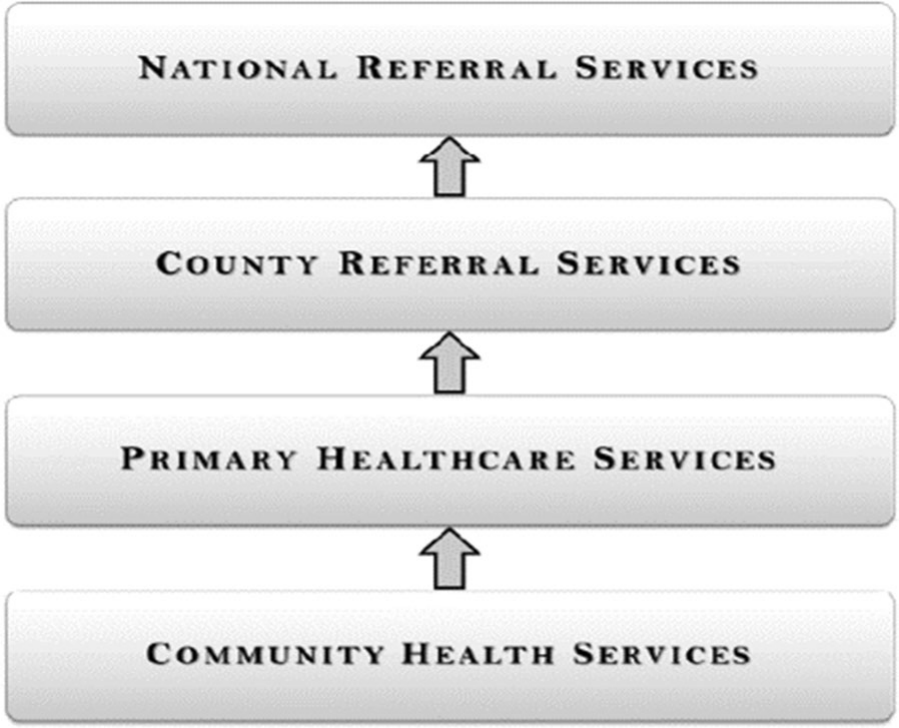
Organization of service provision in the Kenyan public health sector

Kenya’s health care delivery system is organised in a four-tier system (Fig. 1). The lowest tier is community health services (CHS), which includes community health units (CHUs) and involves the creation of demand for services, promoting healthy behaviours, community diagnosis, management and identifying cases within communities that need referral to higher levels of care. The second tier comprises primary care facilities and includes dispensaries, health centres, private clinics and maternity homes. The primary care facilities are tasked with providing promotive, preventive, essential outpatient curative services, emergency inpatient services and facilitation of referrals from the community to the referral facilities. The third tier comprises sub-county and county referral hospitals operated and managed by a given county and act as the first referral level. They provide comprehensive inpatient diagnostic, medical, surgical, rehabilitative and reproductive health services, and specialized outpatient services, and facilitate and manage referrals from lower and other levels. The fourth, top-most tier is the national referral facilities, which provide highly specialized services and include all tertiary referral hospitals, national laboratories, and services, research and training institutions [7].

In the past, the delivery of PHC services in Kenya was characterized by a top–bottom approach that designed and implemented health programmes decided at the national and county levels and a vertical approach that implemented disease-specific programmes without community participation, which compromised the quality of care [8]. Moreover, although PHC services should be offered through public and private health centres, dispensaries, maternity homes and community health services, they are provided across all categories of health facilities, including higher-level facilities. The Ministry of Health (MoH) developed the Kenya Primary Health Care Strategic Framework (PHCSF) 2019–2024 to ensure quality PHC for all citizens in response to the call of the Astana Declaration 2018 [8]. The framework proposes several PHC strategic objectives and interventions regarding the provision of health care services, leadership and governance, drugs and other medical supplies, financing of PHC delivery and the roles of each of the main stakeholders involved, particularly communities. One of the implementation mechanisms of the framework is the establishment of primary care networks (PCNs) and accompanying guidelines that provide for the establishment, operation and management of PCNs within the country [9].

A PCN is a group of health care facilities, deliberately interconnected through an administrative and clinical management model and collaboratively work to provide integrated PHC to the general population [10–12]. PCNs are a form of network of care (NOC) that was initially developed and implemented across some low- and middle-income countries (LMICs) to improve maternal and newborn health care [12–19]. Reports across these countries indicate that NOCs were associated with a reduction in maternal, neonatal and perinatal mortality [12–15, 17–19], and they reduced overcrowding at referral hospitals by decongesting higher-level facilities [14, 19].

PCNs have been implemented in some high-income countries since the early 2000s to integrate primary care to improve population health at the community level [10, 11, 20–22]. Evidence from these countries suggests that PCNs can potentially reduce the risk of hospital admission or visits to emergency departments and improve the quality of primary care and clinical outcomes. Additionally, they can enable better coordination of services and enhance financial and workforce sustainability within primary care [11, 20, 21, 23]. Lastly, PCNs are increasingly being implemented in LMICs, with the evidence indicating a reduction in maternal and child mortality, improved referrals and greater service availability and readiness [12–19, 24].

While PCNs have been implemented in high-income countries [10, 11, 22, 25] and some LMICs [12–19], experience in LMICs is still limited. Specifically, evidence is needed on the design, implementation arrangements, and experiences of PCN reforms, political economy, as well as the impact of these reforms to inform the refinement of the design and implementation of PCNs. Further, health systems and health system reforms are gendered, with implications for equity on both the supply and demand side of health systems [26]. Understanding the gendered implications of PCNs is therefore critical. This proposed work aims to assess the impact, implementation experience, political economy and gendered implications of PCNs in Kenya.

### Primary care networks (PCNs) in Kenya

**Fig. 2:**
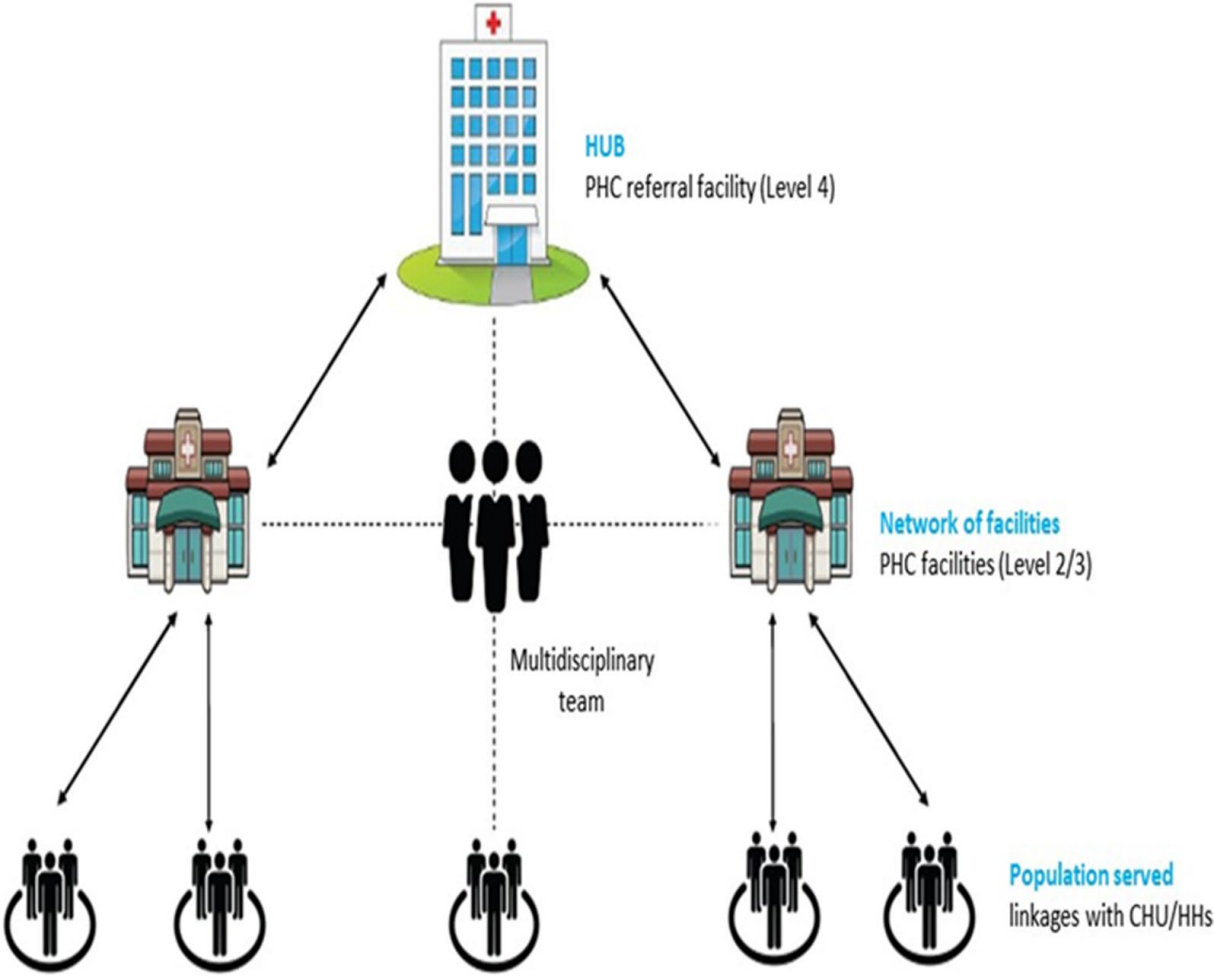
Proposed model of the primary health care network: the hub and spoke model [9]

Since 2020, the Kenya MoH has established primary care networks (PCNs) at the sub-county level as a configuration made up of a PHC referral facility and several other PHC facilities where a level 4 facility serves as the hub and level 2 and 3 facilities are spokes (Fig. 2) [8, 9]. The hubs are the first level of referral in the counties and should provide technical and supply support to the spokes. The main goal of establishing PCNs was to increase the efficiency and effectiveness of health care services with the main objective of improving the coordination of care to ensure patients receive the right care in the right place at the right time at all levels of care [9]. In 2023, the Kenyan government passed the Primary Health Care Act 2023, which seeks to introduce and govern PCNs as part of the national rollout initiative [27].

The national MoH supports the counties in establishing and monitoring PCNs, while the counties are responsible for implementing and coordinating PCN activities. A multi-disciplinary team (MDT), led by a family physician with a mix of different health professional expertise and skills, will oversee the daily activities of the hub, as well as linkages with the spokes and level 1 units within a PCN. A PCN should include at least one level 4 health facility (public, private or faith-based), three level 2 or level 3 health facilities within the region (public, private and faith-based) and five level 1 CHUs. Before establishing a PCN, the geographic area, distance, population size, availability of different cadres of health care workers, adequate financial resources, facility infrastructure and availability of commodities should be determined to inform the establishment of the governance and coordination structures, and mapping of the hubs, spokes and CHUs. A functional PCN is expected to have a PCN coordinator, a coordinated referral system, clearly defined linkages between the hub, spokes and CHUs, a functioning system for logistics, and established monitoring and reporting systems and to be gazetted in the respective county gazette [9].

**Fig. 3:**
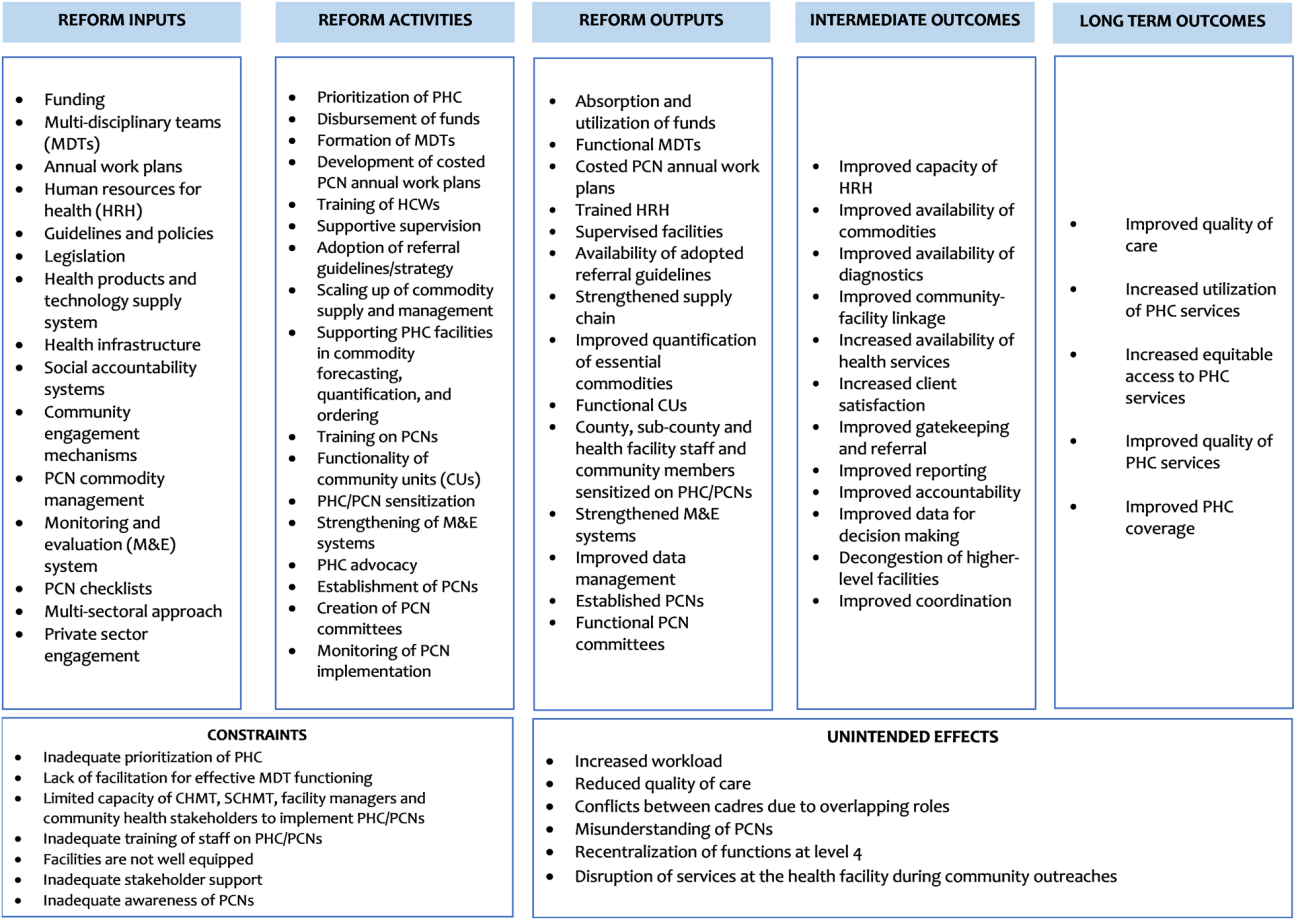
PCN theory of change

Each PCN is responsible for providing and ensuring continuous and uninterrupted essential PHC services while utilizing resources available within its geographical region. The resources can include reorganizing human resources, infrastructure, health products and technologies, finance and governance structures to be responsive to the community’s health care needs [9]. Table 1 outlines how PCNs are expected to operate within the existing health system according to the health system building blocks and are the aspects we shall consider in the process evaluation, as highlighted in the study’s theory of change in Fig. 3.

**Table 1:** PCN operation in Kenya

Health system domain	What PCNs are doing differently
1. Leadership/governance	• Development of PCN annual work plans • The referral facility (hub) manages PHC facilities (spokes) through multi-disciplinary teams (MDTs), which consist of a mix of experts and skills at the hub level
2. Health care financing	• No changes
3. Health workforce	• Capacity building of health care workers at the hub level • Shared human resources – the MDTs through mentorship and supportive supervision of the PHC facilities
4. Medical products and technologies	• Strengthen the supply chain for essential commodities • Share laboratory and radiological services through networking, commodity support and reporting systems • The hub is expected to support the spokes in forecasting, quantifying and ordering commodities
5. Service delivery	• The hub is to coordinate with spokes to define the catchment population for each PHC facility, which should be 100% covered by CHUs linked to the PHC facilities • Joint identification of priority health needs with the community • MDTs to expand the range of services provided at the PHC facilities through regular outreaches • Coordinated referral of patients
6. Information and research	• Strengthening of community-based health information system (CHIS)

### Implementation status

The national target is to have at least one PCN per sub-county (total of 315). By now, 205 (65%) PCNs have been fully established and supported by the Transforming Health Systems (THS) World Bank Project, United States Agency for International Development (USAID), United Nations International Children's Emergency Fund (UNICEF), Amref Africa, Program for Appropriate Technology in Health (PATH), and other partners.

### Study objectives

The specific objectives of this study are:

1. To assess the impact of PCNs on PHC functioning and health service delivery
2. To assess the implementation experience of PCNs
3. To assess the gendered implications of PCNs in Kenya
4. To undertake a political economy analysis of the PCN reforms in Kenya

## Methods

### Theory of change

This evaluation will be guided by a theory of change (ToC), which was adapted from the MoH’s PCN logic model [9] and refined in consultation with stakeholders at a co-creation workshop held in April 2023 that brought together 25 health sector stakeholders drawn from the national- and county-level governments and development partners. The ToC outlines how inputs, which are resources available to implement the PCNs, are converted to outputs through the various activities that the implementers should undertake. It also shows the intermediate- and long-term results to be achieved once the PCNs are functional and effective given the various assumptions, ultimately contributing to a reduction in morbidity and mortality resulting in UHC (Fig. 3).

The goal of PCNs is to increase the efficiency and effectiveness of health care services for patients, particularly those at risk of poor health outcomes [9]. To achieve this, one of the key activities might include allocating funds to the PCNs, forming MDTs and equipping primary health facilities. An important contextual factor might be the policy environment, including the presence or absence of legislation allowing county resources to be allocated to the PCNs and joint ordering of health commodities. Another important activity might be setting up PCNs among CHUs and primary health facilities, which could be affected by local contextual factors such as the sub-county and health facility administration, the nature of CHUs and the willingness to work collaboratively. Some assumptions might include that the counties will adopt the guidelines, allocate funds to PCNs, and train facility managers and the communities on PCNs, that health facilities, health care workers and CHUs will join the network and work collaboratively, and that there will be a favourable policy and political environment. This study will focus on the various outcomes (indicators) from the inputs to outcome sections of the theory of change described in the study design section below.

### Study setting

We will purposively select eight intervention and four control counties. The intervention counties will be selected from a list of counties that are already implementing PCNs (four counties) and counties that are planning to implement PCNs (four counties), considering geographical spread and heterogeneity in funders/partners supporting the establishment of the PCNs. The comparison counties (four counties) will be selected from a list of counties with comparable characteristics, including the region of the implementing counties.

### Indicators and data sources

The primary outcome at the health facility level is health services readiness, while at the client level it is satisfaction with available PHC services. Table 2 outlines the primary and secondary outcomes and the data sources for the evaluation.

**Table 2:** PCN outcome measures and the data sources

Outcome measure	Indicator	Definition	Type	Data source
Objective 1: Assessing the impact of PCNs on PHC functioning and health service delivery				
Health facility readiness and service availability	Availability of essential medicines	Percentage of health facilities with available non-expired essential medicines among the assessed HFs	Quantitative	Health facility assessment (HFA) survey
	Stockouts of essential commodities	Percentage of facilities with stockouts in the past 3 months among the assessed HFs	Quantitative	HFA survey
	Availability of basic equipment	Percentage of health facilities with available basic equipment among the assessed HFs	Quantitative	HFA survey
	Availability of tracer diagnostics	Percentage of health facilities with diagnostic services among the assessed HFs	Quantitative	HFA survey
	Availability of basic amenities	Percentage of health facilities with basic amenities among the assessed HFs	Quantitative	HFA survey
	Improved medical records	Percentage of health facilities using electronic health records among the assessed HFs	Quantitative	HFA survey
	Availability of referral guidelines	Percentage of health facilities with adopted referral guidelines among the assessed HFs	Quantitative	HFA survey
	Provider caseload	Case load per provider per day	Quantitative	HFA survey
	Health information system	Percentage of health facilities capturing data in any HMIS among the assessed HFs	Quantitative	HFA survey
Human resources for health (HRH)	Availability of health workers	Percentage of available health workers by cadre as per the norms and standards	Quantitative	HFA survey
	Absence rate	Percentage of health workers absent from the facility on the day of the survey	Quantitative	HFA survey
Access to health services	Utilization of health services	Number of OPD visits	Quantitative	Kenya Health Information System (KHIS)
	Maternal deliveries	Number of maternal deliveries	Quantitative	KHIS
	ANC visits	Number of ANC visits	Quantitative	KHIS
	DPT3 vaccination	Number of children that received DPT3 vaccine (under 1)	Quantitative	KHIS
	Full immunization	Number of infants that are fully immunized	Quantitative	KHIS
	BCG vaccination	Number of children that received BCG vaccine (under 1)	Quantitative	KHIS
	Clients who received the required services	Percentage of clients who received the required services	Quantitative	Client exit survey
	Transportation	Means of transport	Quantitative	Client exit survey
	Time taken to reach health facility	Time in minutes to reach the health facility from home	Quantitative	Client exit survey
	Expenditure on health care	Amount of out-of-pocket expenditure	Quantitative	Client exit survey
Gatekeeping	Clients whose first contact with the health system was at level 1, 2 or 3	Percentage of clients whose first point of contact with the health system was level 1, 2 or 3	Q uantitative	Client exit survey
Referral	Referral patterns	Percentage of clients who were referred	Quantitative	Client exit survey
		Number of patients referred to other facilities	Quantitative	KHIS
		Number of patients referred from other facilities	Quantitative	KHIS
Decongestion	Patient waiting time at level 4 facilities	The average waiting time	Quantitative	Client exit survey
	Client perception of waiting time	Percentage of clients who perceived the waiting time to be alright	Quantitative	Client exit survey
Quality of care	Client-reported experiences with care	Percentage of clients satisfied with the available PHC services	Quantitative	Client exit survey
	Client satisfaction with quality of care	Percentage of clients satisfied with the quality of available PHC services	Quantitative	Client exit survey
	Timely access to care	Percentage of clients reporting that they receive the care they needed in a timely manner	Quantitative	Client exit survey
Health facility-community linkage	CHU linkage to HF	Number of functional CHUs linked to HF	Quantitative	KHIS/HFA survey
		Number of clients referred from the community	Quantitative	Client exit survey
		Number of clients that have been referred to the facility by community health volunteers/promoters	Quantitative	KHIS
Objective 2: Assessing the implementation experience of PCNs				
Process evaluation	Finances	Proportion of county budgetary allocation to PCNs, funding flows and spending	Quantitative	County budget reports
	Availability of implementation plans	Number of costed PCN work plan per sub-county	Quantitative	Sub-County Medical Officer of Health (SCMOH) reports
Leadership, governance and coordination	PCN Advisory Council meetings	County PCN Advisory Council meetings	Quantitative	County director of health (CDH) reports
	Inter-sectoral and partnership forum meetings	County PCN inter-sectoral and partnership forum meetings	Quantitative	CDH reports
	Inter-sectoral representatives’ forum meeting	Sub-county PCN inter-sectoral representatives’ forum meetings	Quantitative	SCMOH/MDT – lead reports
	PHC TWG meetings	Number of quarterly meetings held by the PHC TWG	Quantitative	SCMOH
	Coordination	A description of coordination mechanisms	Qualitative	In-depth interviews
		Presence of a coordinated referral system with back-and-forth documentation	Qualitative	In-depth interviews
HRH	PCN sensitization	Number of CHMT members sensitized on PCNs	Quantitative	County PCN report
		Number of health facility managers sensitized on PCNs	Quantitative	County PCN report
	Creation of MDTs	MDT lead and team members identified	Quantitative	County PCN report
	PCN training	Annual report on training conducted for the Community Unit Workforce	Quantitative	County PCN report
	Multidisciplinary team-based service delivery	Services provided by the MDTs	Quantitative	HFA survey
	Functional community health units (CHUs)	The number of CHUs meeting the criteria for a functional CHU defined in the guidelines and linked to a health facility	Quantitative	KHIS/HFA survey
	Functional PCNs	The number of PCNs meeting the criteria for a functional PCN	Quantitative	County PCN reports
	Functional MDTs	The number of functional MDTs	Quantitative	County PCN reports
	PCN training	The proportion of trained health workers by cadre	Quantitative	HFA survey
	Implementation experience	A description of the implementation experience and understanding of PCNs by stakeholders	Qualitative	In-depth interviews
		A description of the effect of PCNs on workload, quality of care, efficiency, relationships between cadres/leadership and autonomy of HFs	Qualitative	In-depth interviews
	Community engagement	A description of community engagement in service planning and organization	Qualitative	County PCN reports
Objective 2: Assessing the political economy of PCN reforms				
	Stakeholders and their interests	A list of all the actors, including the government and implementing partners and their roles, actions, interests, influence and power relations	Qualitative	Document reviews/in-depth interviews
	Power analysis	A description of actor interests, roles, level of power, influences and power relations	Qualitative	Document reviews/in-depth interviews
Objective 4: Assessing the gendered implications of PCNs				
	Leadership by gender and the implications	A description of top and facility leadership by gender and how it affects implementation of PHC	Qualitative	In-depth interviews
	Service utilization by gender	Sub-analysis of outpatient visits by gender	Quantitative	KHIS
	MDT members by gender	The composition of MDT by gender and how it affects service delivery	Quantitative and qualitative	HFA and in-depth interviews
	Access by gender	Sub-analysis of clients who received the required services by gender	Quantitative	Client exit survey

### Study design

We will adopt the parallel-databases variant of the convergent mixed methods design, where we will concurrently but separately collect quantitative and qualitative data [28]. The convergent parallel mixed methods design integrates quantitative and qualitative data collected independently but simultaneously to provide a comprehensive understanding of PCN implementation. By triangulating measurable outcomes with contextual insights, this approach enhances validity, uncovers the “what” and “why/how”, and ensures findings are robust, relevant and applicable to policy and practice. Both data types will hold equal importance in addressing the study’s research questions.

This will be followed by analysis of the two datasets separately and independently using standard quantitative and qualitative analytic procedures. After obtaining the initial results, we will reach the point of interface, where the findings will be merged. This merging may involve directly comparing the results or transforming them to facilitate integration during further analysis. Finally, we will interpret how the two sets of results converge, diverge or complement each other to provide a deeper understanding of PCN implementation.

**Fig. 4:**
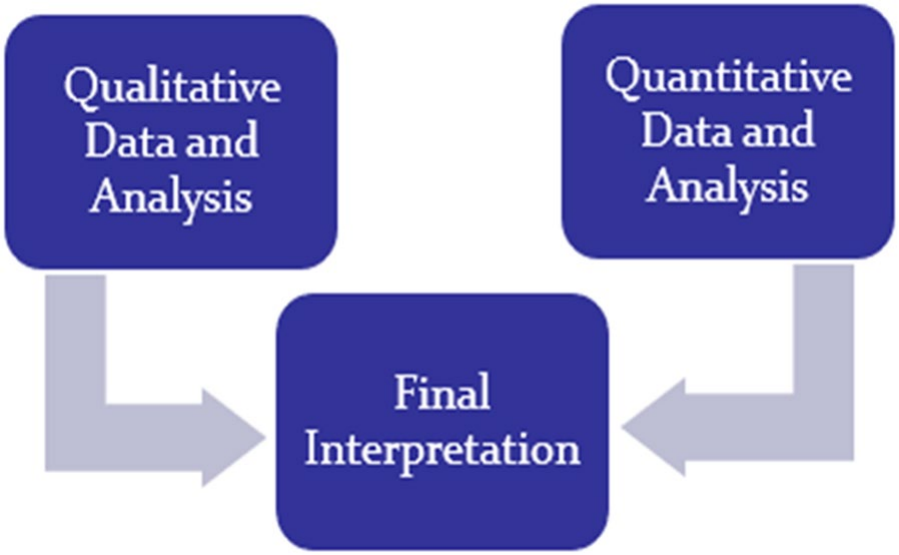
Mixed methods design

Mixing will occur during data collection, with early quantitative findings informing the refinement of interview questions. It will also take place during analysis and interpretation, where qualitative insights into context and mechanisms will enhance the quantitative outcomes for a comprehensive understanding [28, 29]. Data collection will involve a combination of structured questionnaires, semi-structured questionnaires, in-depth interviews and desk document reviews (Fig. 4).

### Quantitative study

#### Study design

The quantitative study will be used to answer objective 1 in evaluating the impact of PCNs on PHC functioning and service delivery at primary care facilities and objective 4 in assessing the gendered effects of PCNs in Kenya using a controlled before and after study design as our primary design. Our key evaluation question will be: what is the impact of PCNs on PHC functioning and service delivery readiness? We will undertake a prospective impact evaluation by identifying the intervention and control counties and collecting the baseline data before the PCNs are implemented and 12 months after implementation.

#### Study population

Before and after cross-sectional health facility and client surveys will be conducted. For each survey, the study populations will include a random sample of 228 health facilities and 2560 clients in four intervention (currently implementing), four intervention (planning to implement) and four control counties at baseline and post-implementation. The inclusion and exclusion criteria for each study population are specified below:

#### Inclusion criteria

Inclusion criteria for health facilities are:

- Primary care facilities owned by the government, private sector, non-government or faith-based organisations
- Health facility providing general outpatient services for all age groups

Inclusion criteria for clients are:

- Clients presenting for an outpatient visit on the survey day

#### Exclusion criteria

Exclusion criteria for health facilities are:

- Health facilities providing only specialised health services (e.g. tuberculosis (TB) clinic)
- Tertiary hospitals serving mainly as national referral facilities

Exclusion criteria for clients are:

- Clients admitted/requiring admission
- Clients with surgical illnesses

#### Sample size and sampling

There will be three levels of sampling for this study. The first level will be purposive sampling of counties into three groups: (1) those already implementing PCNs, (2) those planning to implement the PCNs and (3) those not implementing as control. We will classify all 47 counties into three types: those already implementing PCNs, those planning to implement and those not implementing. We will purposively select four counties that have recently implemented PCNs, and four that are most advanced in their plans to implement PCNs as intervention counties from the list of counties already implementing and those planning to implement PCNs together with the MoH and county representatives. This will be followed by a purposive selection of four control counties that are most comparable to the selected intervention counties in terms of socioeconomic status, geographical location and population.

Across the selected counties (Table 3), we will randomly sample 76 health facilities per group, assuming a mean difference of 10% in the utilization of health services between the groups and a population variance of 400 across the groups using 80% power, 95% confidence level and a design effect of 1.2, for the health facility assessments. The health facilities will include level 4 (county, sub-county or faith-based hospitals), 3 (health centres) and 2 (dispensaries) facilities. In intervention counties, we will sample at least four health facilities (one hub and three spokes) as recommended by the guidelines per PCN. In PCNs with less than the recommended number of health facilities, we will include all health facilities in the sample. For those with more than four facilities, we will proportionally sample them. We will proportionally sample level 4, 3 and 2 facilities in control counties.

**Table 3:** Characteristics of sampled counties

County	Population (2019 census)	Level 5 facilities	Level 4 facilities	Level 3 facilities	Level 2 facilities	Total
Implementing PCNs						
Garissa	835 482	1	10	23	55	89
Kisumu	1 144 777	1	25	50	79	155
Kwale	858 748	0	4	12	110	126
Nakuru	2 142 667	1	18	43	176	238
Planning to implement PCNs						
Kakamega	1 861 332	1	17	57	120	195
Makueni	977 015	0	15	39	210	264
Busia	886 856	0	8	17	73	98
Lamu	143 920	0	3	5	29	37
Control counties						
Nyandarua	636 002	0	3	24	67	94
Homabay	1 125 823	0	20	64	146	230
Kajiado	1 107 296	0	7	32	89	128
Tana River	315 943	0	3	4	56	63

For the client exit interviews, we will seek to interview clients who received at least one service from the 228 facilities. We will use a random sample of 924 clients exiting the health facilities per group, assuming a mean difference of 5% in client satisfaction between the two groups and a population variance of 1225 across the groups using 80% power, 95% confidence level and a design effect of 1.2. A minimum of 20 clients per facility will be interviewed. The following sample size calculation formula was used:

$$n=\left({{Z}_{\alpha /2}+Z}_{\beta }\right)sqrd*2* {\upsigma }^{2} /{d}^{2}$$

where:

*Z*_*α*/2_ =1.96 (5% significance)

*Z*_β_ = 0.84 (80% power)

*σ*^2^ = population variance of 1225 (standard deviation of 35 per group (based on the number questions (*n* = 35) with 35 possible worst response based on a 5-Likert scale), and

*d* = mean difference of 5% between the groups.

#### Data collection

The data collection methods will include both secondary and primary data collection across implementing and control counties using the same tools. For the secondary data, we will extract data on a list of indicators from the Kenya Health Information System (KHIS) to include 12 months before the introduction of PCNs and the first 12 months of its implementation.

Primary data will be collected using structured electronic questionnaires in REDCap software by research assistants trained before each survey. The data will be collected at baseline and 12 months after the implementation of PCNs. At each health facility, data will be collected over three days using two methods. First, all patients presenting to the outpatient departments on the survey day will be screened for eligibility when they are ready to leave the facility and included in the study if they meet the inclusion criteria and provide informed consent.

Information on their characteristics, socio-economic status, services sought, whether they received the services, and their perspective on the quality of services, facility access, professional competence, medical costs, efficiency, facility infrastructure, availability of drugs and services, health worker attitude, and satisfaction with services will be collected using a client exit survey tool.

Second, to assess the health facility readiness to provide PHC services, we will conduct facility assessment that will include interviews with facility in-charges and direct observations to determine the availability of essential drugs, basic equipment, facility profile, target population, services provided, availability of laboratory services and service utilization using a health facility assessment tool.

Lastly, to assess the gendered effects of PCNs, we will collect data on the gender of facility in-charges, MDT team leads and board chairpersons. Additionally, we will examine the gender composition of committees, boards or MDT members, as well as outpatient utilization rates disaggregated by gender.

#### Data analysis

The data will be exported from REDCap software in Excel format and imported into STATA for analysis. The unit of analysis will be the health facilities and clients. We will first conduct a descriptive analysis of data – means and proportions – to explore and summarize the data at baseline and endline. This will be followed by a preliminary cross-sectional analysis of the data at baseline comparing the differences in outcomes between intervention facilities (the four already implementing) and the four control counties. Lastly, we will conduct a difference in difference regression analysis at endline comparing differences in outcomes between the intervention (four intervention counties planning to implement) and four control counties to measure the impact of PCNs on service utilization and service readiness (health commodities availability, financial resources availability, human resource availability etc.).

The difference-in-differences method compares the changes in outcomes over time between a population that is enrolled in a program (the treatment group) and a population that is not (the comparison group) because counties can choose to implement or not implement PCNs. The facilities and clients from the eight intervention counties will serve as the treatment group, and the others from the four control counties as the comparison group.

For the health facility-level analysis, we will control for the level and type of facility and county-level characteristics such as population and socioeconomic status, adjusting for clustering at the sub-county level. In the client-level analysis, in addition to controlling for the above county- and health facility-level factors, we will also control for client characteristics such as age, gender, level of education and marital status, adjusting for clustering at the health facility and sub-county levels. All analysis will be undertaken in STATA.

### Qualitative study

#### Study design

The qualitative study design, including process evaluation and political economy analysis, will be used to answer objectives 2, 3 and 4. We will undertake a cross-sectional qualitative study to (1) assess the fidelity and implementation experience of PCNs, (2) examine the political economy of PCNs and (3) explore the gendered effects of the PCNs.

#### Conceptual framework

**Fig. 5:**
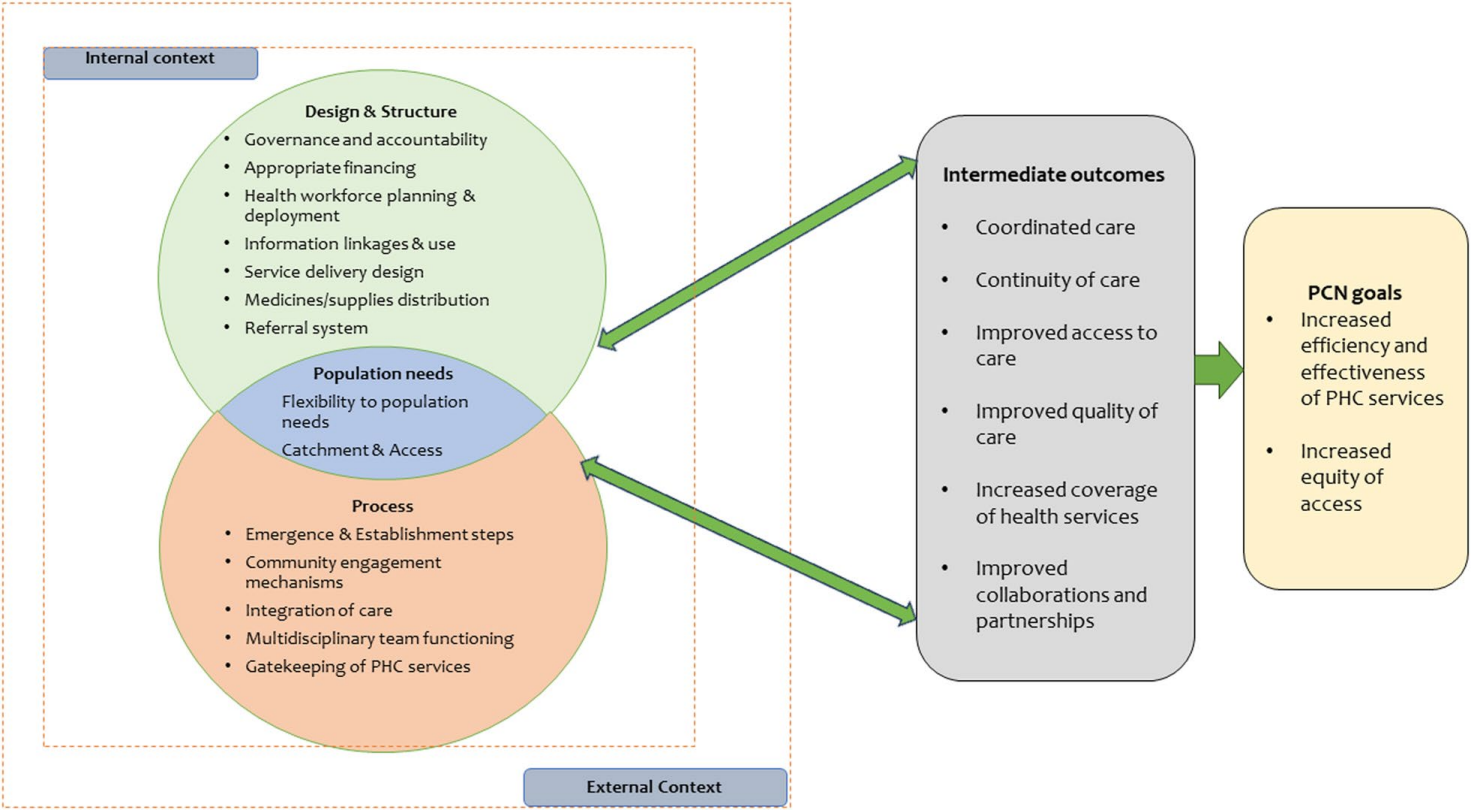
Qualitative conceptual framework

This study’s qualitative framework was informed by various frameworks for evaluating health networks [12, 14, 30]. This framework is grounded on the understanding that PCNs comprise three key dimensions; the structure of the health system including the six building blocks and process of implementation interacting with population needs influenced by external and internal context of the health system such as health policy and regulations to achieve the PCN goals (Fig. 5). We will use this framework to develop interview guides that will explore the process evaluation and examine the political economy and the gendered effects of PCNs.

#### Process evaluation

The process evaluation of the PCNs will aim to capture the emergence of PCN reforms, how they were implemented, the mechanism of impact and how the context affects implementation and outcomes. For the emergence of PCN reforms, we will examine the factors that led to the reform and the adopted approaches. For the implementation, we will assess the fidelity of the PCNs, whether they were implemented as intended by the guidelines, how they were delivered and whether they are reaching the intended population and how and the unintended effects of PCNs. For the mechanism of impact, we will assess the mechanisms through which PCNs bring change in PHC delivery. Lastly, we will assess the socioeconomic and political factors that might hinder or facilitate PCN implementation [31]. The process evaluation will include the following elements drawn from the theory of change, and the data will be collected through document reviews and in-depth interviews with key informants (Table 4).

**Table 4:** PCN process evaluation outcomes

Specific objective	Qualitative indicators
Emergence of PCN reforms	What challenges necessitated the PCN reforms?
	What options existed for resolving the challenges?
	What was the basis for the adopted way of resolving the challenges?
Fidelity of the implementation process	Description of the implementation as required by the guidelines
	Description of how PCNs are implemented on the ground
Implementation process	Actors’ experiences of implementing the PCNs
	Adaptations triggered by the implementation of PCNs
	Unintended consequences of PCNs
	Contextual issues that influence the PCNs
Explanation of outcomes or outputs	Reasons for the observed quantitative results

#### Political economy analysis

**Fig. 6:**
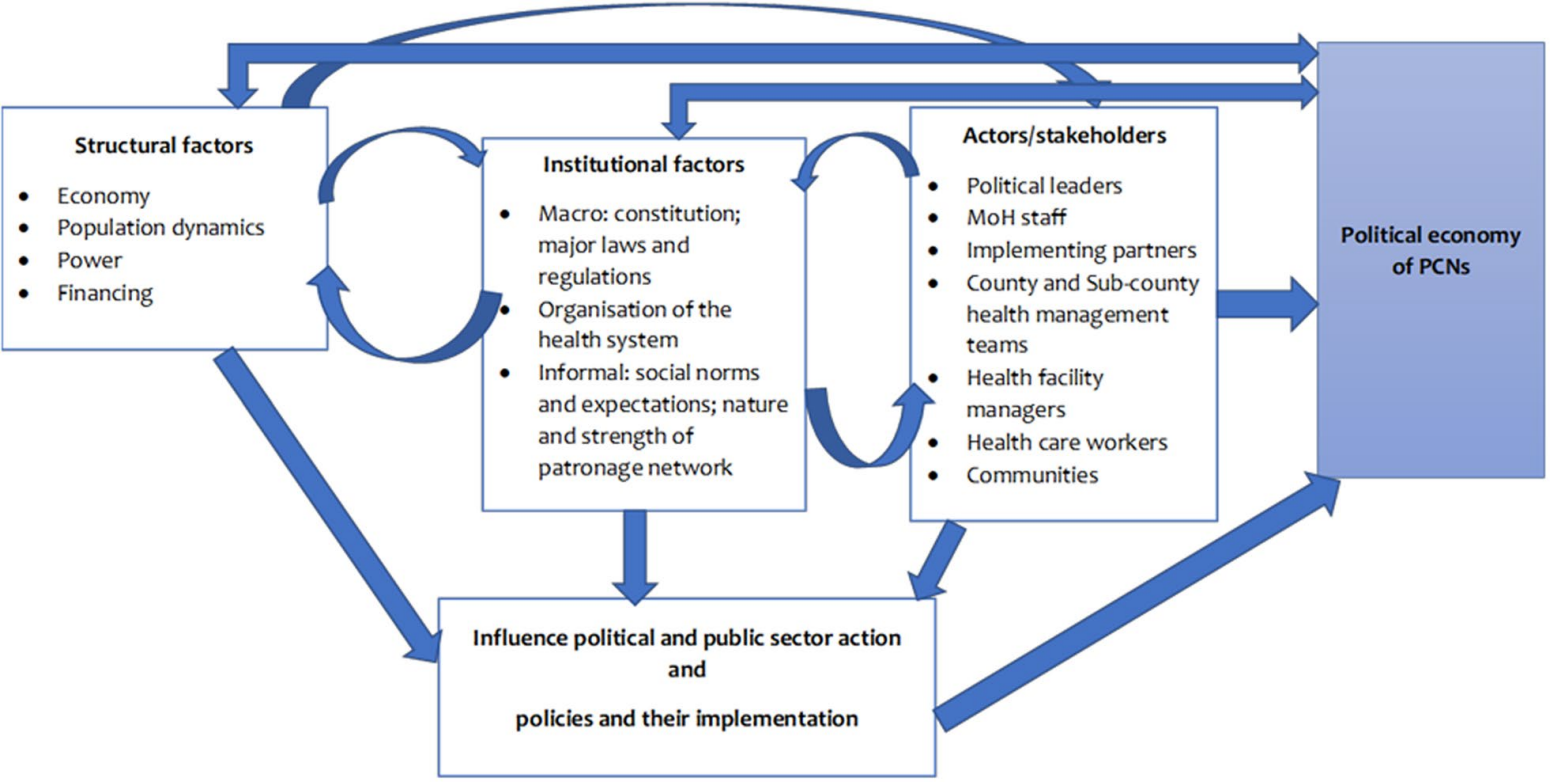
PEA framework for PCNs [32]

The political economy analysis (PEA) will be exploratory and will examine the interaction of structural factors, institutions and actors/stakeholders’ interests and power relations in the implementation of PCNs using the framework in Fig. 6. We propose to use a modified problem-driven PEA [32]. The problem-driven political economy analysis has three aspects: (i) description of the problem; (ii) institutional analysis, which entails mapping out all the stakeholders involved in implementing PCNs, and the legal and policy documents that relate to the problem; and finally (iii) identification of political economy drivers – specifically this will entail describing why things exist the way they do [32]. In exploring the drivers, we will consider three structures of drivers: structural (economy, population dynamics, power), institutional (rules and regulations, organization of the health system) and actors/stakeholders (government and non-government) (Fig. 6) [32].

#### Gendered effects of PCNs

We will assess the gendered implications of PCNs both quantitatively and qualitatively. We will use the gender analysis framework proposed by Morgan et al. [33], which argues that gender is a power relation and driver of inequity in health systems and can be understood by how power is constituted and negotiated. We will focus on four key domains by asking who has what (access to resources), who does what (the division of labour and everyday practices), how values are defined (social norms, ideologies, beliefs and perceptions) and who decides (rules and decision-making) [33] across the six health system building blocks (Table 5).

**Table 5:** Gender as a power relation and driver of inequity in health [33]

What constitutes gendered power relations		
Who has what	Access to resources (education, information, skills, income, employment, services, benefits, time, space, social capital etc.)	
Who does what	Division of labour within and beyond the everyday practices	
How are values defined	Social norms, ideologies, beliefs and perceptions	
Who decides	Rules and decision-making (both formal and informal)	
How power is negotiated and changed Individual/People	Critical consciousness, acknowledgement/lack of acknowledgement, agency/apathy, interests, historical and lived experiences, resistance or violence	
Structural/environment	Legal and policy status, institutionalisation within planning and programs, funding, accountability mechanisms	

We will quantitatively assess the services available at the health facilities, the gender composition of the facility management, leadership and composition of the MDTs. Moreover, we will conduct a sub-analysis of outpatient visits from the KHIS, and client exit data focusing on their education level, services received, out-of-pocket payments and insurance coverage by gender. We will complement the quantitative findings with qualitative inquiry on a description of top leadership by gender and how it affects implementation of PHC and the composition of MDT by gender and how it affects service delivery. The qualitative inquiry will be included in both the process evaluation and PEA, where we will assess the gender of who makes and implements decisions and the gender distribution of stakeholders.

#### Study population

Stakeholders from the national, county, health facility and community levels will be interviewed.

#### Sample size and sampling

We will sample national, county, facility and community stakeholders involved in PHC service delivery decision-making, implementation and delivery for the qualitative interviews. On the basis of the number of people holding these positions, we anticipate conducting 5–6 interviews at the national level and 9–15 interviews from each county (Table 6). However, the final numbers to be included in the interviews will depend on the level of data saturation.

**Table 6:** Participants to be sampled for the qualitative study

Participant category	Subcategory	Number of participants
National level participants	National Treasury	1
	Ministry of Health	3
	Development partners	6
	Implementing partners	4
	Council of governors	1
County-level participants (per county)	County Treasury	1 per county, a total of 5
	County Department of Health	6 per county, a total of 30
	Sub-county managers	1 per county, a total of 5
	Health facility managers	2 per county, a total of 10
	Frontline health workers	1 per county, a total of 5
	Community health committee members	1 per county, a total of 5
	Community health promoters	1 per county, a total of 5
Total participants		80

#### Data collection

We will use semi-structured interview guides to collect the qualitative data. We will begin by conducting a desk review of county PCN reports, guidelines, strategic plans, reports from partners and PCN review reports to describe the PHC policy context and implementation of PCNs in Kenya. This will be followed by qualitative stakeholder in-depth, semi-structured interviews. The participants will be provided with an information sheet, and consent will be sought for participation in the interviews. The interviews will be conducted face-to-face, audio recorded and transcribed verbatim. The semi-structured and open-ended interview questions will explore participants’ understanding of the purpose of the PCNs, gauge how they see PCNs fulfilling that purpose and illuminate any barriers and opportunities they have encountered in establishing PCNs. We will also explore with the interviewees how PCN facilities integrate the delivery of care, how they work both vertically and horizontally within the health system, how the design and operational features (including their governance, leadership and management, financing, human resource management, procurement etc.) influence the feasibility of implementation and effectiveness of PCNs. Lastly, we will explore and map out actors’ interests in PCNs and their power relations.

#### Data analysis

The interview transcripts will be imported into in NVivo (version 12) for coding and analysis using a thematic approach. The first step in this analysis process will be familiarization with the interviews by reading interview transcripts and/or listening to audio recordings. Herein begins the process of abstraction and conceptualisation of the data. Second, after familiarization, we will apply labels or codes in the passage we interpreted as important and relevant. Codes will be developed inductively and deductively. Deductive coding will be guided by constructs in the conceptual framework presented in Fig. 5. The researchers will ensure inter-coder agreement by conducting a pilot coding. Two researchers will code a subset of the transcripts independently. These codes will then be compared, and discrepancies in the coding system will be discussed and refined appropriately, resulting in a final coding framework.

The coding framework will then be applied to all the remaining transcripts. Third, similar codes will be grouped into categories or themes which are clearly defined to form a working analytic framework. The framework will draw upon (a) a priori issues (those informed by the original research aims and introduced into the interviews via the topic guides), (b) emergent issues raised by the respondents themselves and (c) analytical themes arising from the recurrence of particular views or experiences that are interpreted to be important and relevant. The fourth step will involve charting the data into the framework matrix developed using the NVivo software. Charting will allow the development of a summary of the data by category from each of the transcripts and move into the last step of the framework analysis and interpretation. This approach will provide findings and interpretations that will be triangulated with quantitative results relevant to policy and provide pragmatic recommendations. We will present the implementation process, political economy and gendered implications of PCNs, as outlined in the specified analysis areas and outcomes.

#### Validity and reliability of study instruments, rigour and trustworthiness

Validity of the quantitative data will be ensured through the study process from study design, sampling, development of tools, data collection and analysis. We will ensure that the quantitative data collection tools elicit all the information required to answer the questions on the impact evaluation of PCNs. As we have discussed, indicators must be measured throughout the theory of change, including indicators for intermediate- and long-term outcomes. At the same time, the qualitative data tools must capture information on the emergence of reforms, the fidelity of implementation, implementation experience, the factors influencing implementation, actors’ interests and power relations.

During data collection, the validity of the data will be ensured through a 3-day training of research assistants on study methodology, tools and responsible conduct of the study using standard operating procedures that will also be used throughout fieldwork. Moreover, the data collection will be supervised, and the collected data will be checked for any errors at the end of each survey day. The data collection tool will also have data quality checks to ensure the right data is collected. Data analysis will be undertaken according to a prepared analysis plan. Lastly, the reliability of the data collection tools will be ensured through pre-testing of the tools during the piloting and consistency in the data collection processes.

We will utilize various strategies throughout the qualitative research process to increase the trustworthiness of the data according to Guba’s [34] four aspects: (a) truth value, (b) applicability, (c) consistency and (d) neutrality. The credibility of the data will be ensured through adequate submersion in the research setting to identify recurrent patterns and verify them. Moreover, we will triangulate the findings with the quantitative ones and share the preliminary findings with the respondents and internal research team to obtain their feedback.

Transferability will be enriched by providing a detailed account of the conduct of the study and other research processes of sampling (for both methods), data collection and analysis. We will also use reflexivity by constantly reflecting on our personal biases, assumptions and values that may influence the research process. Lastly, we will incorporate respondents’ feedback in our final results by sharing the preliminary findings for validation through dissemination workshops.

#### Pilot study

We will pilot the data collection instruments in a county already implementing PCNs, and the findings will inform necessary amendments to the tools and study. During the piloting, we will ensure that the quantitative data collection tools elicit all the information required to answer the questions on the impact evaluation of PCNs, while the qualitative tools capture all the information needed for the process evaluation and political economy analysis.

### Data management

#### Quantitative data

We will ensure data safety, and no identifiable information will be stored with survey responses. Survey responses will be maintained on secure, password-protected servers at Kenya Medical Research Institute (KEMRI)-Wellcome Trust Research Programme (KWTRP). Deidentified data files will be shared with the research team via secure file transfer and will be maintained on password-protected devices. A database capturing data from the questionnaires will be developed in REDCap software in collaboration with the KWTRP Nairobi data team. Data will be checked for errors and imported into STATA for analysis.

#### Qualitative data

Qualitative data from the in-depth interviews will be collected in the form of audio files and field notes. Field notes will be typed into Microsoft Word, and the audio files will be transcribed verbatim into Microsoft Word. These files will then be kept in a secure folder on a password-protected computer and server and backed up in line with the KWTRP data governance policies. To ensure internal verification and validity of the study, we will audiotape the interviews, transcribe them verbatim and conduct frequent random checks for the accuracy and completeness of interview transcripts. The data from the interviews will be given a consistent anonymized label with personal identifiers removed so that research participants cannot be identified from these transcripts alone. A list of interviewees characteristics and demographic details would be kept separately in a Word document. This data will be made accessible to the research team only. The audio files will be destroyed immediately after transcription, but text files will be stored indefinitely for 5 years as per KEMRI policy.

## Discussion

The introduction of PCNs in Kenya aims to increase the efficiency and effectiveness of PHC services with the main objective of improving the coordination of care to ensure that patients receive the right care in the right place at the right time at all levels of care. However, there is limited evidence on their impact and implementation in resource-limited settings and their gendered implications on PHC. This study will contribute to the nascent body of evidence on the impact and implementation of PCNs from an LMIC setting. The study will provide a close examination of the implementation process and contribute to understanding the factors associated with successful implementation and some of the challenges that implementers face, potential unintended consequences, and the effect on service availability and PHC functioning. Moreover, the process evaluation will provide information on how the PCN implementation in Kenya can be refined to enhance its effectiveness and guide the national scale-up. This evidence will also be valuable to other similar settings in informing PCN intervention design and implementation refinements. The evidence from the political economy analysis, including mapping of the actors, their interests and power relations, will inform strategies for stakeholder management to enhance stakeholder buy-in and support for the PCN reform and thus enhance implementation feasibility. Lastly, evidence on the demand- and supply-side gendered effects of PCN reforms will inform strategies to enhance equity in the supply and demand of health care.

This study is not void of challenges and limitations. First, we do not have control over the PCN rollout. The PCNs might be rapidly rolled out, meaning we will not have control counties. This may force us to change our impact evaluation design, for instance, using sub-counties that have not started implementing PCNs as controls. Second, the implementation is non-standardized across the counties as PCN implementation is supported by several partners with different approaches and goals, which might affect our outcomes. To mitigate this, we will purposively sample the intervention counties considering the heterogeneity of partners supporting the establishment of the PCNs. Third, the lack of random intervention assignment is a major weakness of the quasi-experimental design adopted in this study. However, we can still estimate causality since the intervention precedes the measurement of the outcome, we will have controls, and we will seek to control for confounders. Fourth, there might be contamination by the intervention effect, reaching non-implementing sub-counties. We will purposively select non-neighbouring sub-counties as controls. Lastly, unobserved reasons exist why some counties will implement PCNs while others will not. We will control for county characteristics in the difference-in-difference analysis.

## Data Availability

The dataset from this study will not be shared publicly because they will be under the ownership of the KEMRI-Wellcome Trust Research Programme. The data will be available through a formal request process to the KEMRI Institutional Data Access/Ethics Committee. The details of the guidelines can be found on the KEMRI Wellcome website (https://dataverse.harvard.edu/dataverse/kwtrp). Access to data will be provided via the KEMRI Wellcome Data Governance Committee: dgc@kemri-wellcome.org.

## Abbreviations

CHIS: Community-based health information system
CHS: Community health services
CHU: Community health unit
HFA: Health facility assessment
HRH: Human resources for health
KHIS: Kenya Health Information System
KWTRP: KEMRI-Wellcome Trust Research Programme
LMICs: Low- and middle-income countries
MDT: Multi-disciplinary team
MoH: Ministry of Health
NOC: Network of care
PCNs: Primary care networks
PEA: Political economy analysis
PHC: Primary health care
PHCSF: Primary Health Care Strategic Framework
THS-UCP: Transforming Health Systems–Universal Health Care Systems
TOC: Theory of change
UHC: Universal health coverage
